# Advancements in genome editing tools for genetic studies and crop improvement

**DOI:** 10.3389/fpls.2024.1370675

**Published:** 2025-02-03

**Authors:** Asadollah Ahmadikhah, Homa Zarabizadeh, Shahnoush Nayeri, Mohammad Sadegh Abbasi

**Affiliations:** Department of Cellular and Molecular Biology, Faculty of Life Sciences and Biotechnology, Shahid Beheshti University, Tehran, Iran

**Keywords:** CRISPR/Cas9, crop improvement, genome editing, meganuclease, RNA interference, TALEN, ZFNs

## Abstract

The rapid increase in global population poses a significant challenge to food security, compounded by the adverse effects of climate change, which limit crop productivity through both biotic and abiotic stressors. Despite decades of progress in plant breeding and genetic engineering, the development of new crop varieties with desirable agronomic traits remains a time-consuming process. Traditional breeding methods often fall short of addressing the urgent need for improved crop varieties. Genome editing technologies, which enable precise modifications at specific genomic loci, have emerged as powerful tools for enhancing crop traits. These technologies, including RNA interference, Meganucleases, ZFNs, TALENs, and CRISPR/Cas systems, allow for the targeted insertion, deletion, or alteration of DNA fragments, facilitating improvements in traits such as herbicide and insect resistance, nutritional quality, and stress tolerance. Among these, CRISPR/Cas9 stands out for its simplicity, efficiency, and ability to reduce off-target effects, making it a valuable tool in both agricultural biotechnology and plant functional genomics. This review examines the functional mechanisms and applications of various genome editing technologies for crop improvement, highlighting their advantages and limitations. It also explores the ethical considerations associated with genome editing in agriculture and discusses the potential of these technologies to contribute to sustainable food production in the face of growing global challenges.

## 1 Introduction

The global population is projected to reach 10 billion by 2050, which will require an increase in food production by 60 to 100% to meet the needs of this expanding demographic (Chen et al., 2017; Francisco Ribeiro and Camargo Rodriguez, 2020). The second United Nations Sustainable Development Goal (SDG) focuses on eradicating hunger and malnutrition by 2030, while ensuring that everyone has year-round access to adequate and nutritious food (Fanzo, 2019). Despite ongoing initiatives to improve the global food system, agricultural production is currently falling short of the productivity levels necessary to sustain a population of 10 billion by 2050 (Hickey et al., 2019). Additionally, the effects of biotic stressors (such as pests, fungi, bacteria, and viruses) and abiotic stressors (including drought, heat, salinity, and cold) are exacerbated by human activities. These challenges lead to reduced agricultural land, scarce water resources, and increased competition for dwindling resources, significantly impacting the productivity of plant-based food sources during this period (Pandey et al., 2017).

Crop improvement strategies focus on maximizing yield, improving quality, increasing nutritional content, and strengthening resilience to both biotic and abiotic stressors. Genetic improvements in food crops are increasingly recognized as effective strategies to meet the dietary needs of a growing population while ensuring the protection of consumer preferences and health.

The CRISPR-Cas9 system has attracted significant attention due to its wide-ranging applications in plant breeding, facilitating the development of agricultural crops and advancing biological research. Genome editing, in particular, has been investigated to improve characteristics such as, drought tolerance (Shi et al., 2017), and salt tolerance (Zhang et al., 2019) in key crops, including wheat (Chen and Gao, 2014), maize (Barman et al., 2019), and soybean (Liu et al., 2017). Therefore, this review evaluates the role of CRISPR-Cas9 in enhancing the security, quality, and safety of the food supply. Crop production faces challenges such as declining arable land and rapid climatic changes. Traditional breeding is labor-intensive and slow, while genetic engineering (GE) accelerates the development of high-yield, resilient crop varieties. Despite the potential of genetically modified (GM) crops, their adoption is limited due to health and safety concerns (Mao et al., 2019). Innovative plant breeding techniques enhance productivity and superior crop production. Haploid induction (HI) produces homozygous, genetically uniform lines, accelerating breeding. Apomixis allows seed production without fertilization, fixing traits without genetic recombination. Male sterile lines enable controlled hybrid seed production, and self-incompatibility (SI) regulation promotes genetic diversity of crop plants (Gao, 2021). These strategies, combined with GE, precisely modify plant genomes to improve productivity-related traits.

Traditional mutation methods are inefficient, generating non-targeted genome changes that can be harmful. GE introduces precise genome modifications, utilizing sequence-specific nucleases such as homing endonucleases, ZFNs, TALENs, and CRISPR/Cas. Unlike protein-DNA interaction-based nucleases, CRISPR/Cas targets DNA through Watson-Crick base pairing via single-guide RNA (sgRNA) (Voytas and Gao, 2014). These genetic engineering techniques enhance crop traits, contributing to sustainable agriculture and food security by targeting genes involved in productivity.

## 2 Different molecular editing tools

In recent years, various genome editing techniques have emerged. Meganucleases (MNs) were first described by Choulika et al. (1995) and Rong et al. (2002), while transcription activator-like effector nucleases (TALENs) were extensively investigated by Boch et al. (2009); Moscou and Bogdanove (2009); Christian et al. (2010); Deveau et al. (2010); Zhang et al. (2011); Hockemeyer et al. (2011); Reyon et al. (2012), and Sanjana et al. (2012). Zinc finger nucleases (ZFNs) have been explored by Porteus and Baltimore (2003); Miller et al. (2007); Sander et al. (2011), and Wood et al. (2011). The phenomenon of RNA interference (RNAi) was introduced by Fire et al. (1998). Lastly, the RNA-guided CRISPR nuclease system has been extensively studied by Garneau et al. (2010); Horvath and Barrangou (2010); Makarova et al. (2011); Cho et al. (2013); Cong et al. (2013); Jinek et al. (2013), and Mali et al. (2013).

The significance of double-stranded breaks (DSBs) in genome engineering has been well-established, leading to the use of the I-SceI endonuclease from *Saccharomyces cerevisiae*, a natural homing endonuclease (meganuclease), to facilitate genome modifications in somatic cells. Additionally, technologies such as TALENs and ZFNs, which possess endonuclease activity domains capable of generating targeted DSBs at specific genomic loci, have been developed. On the other hand, the Cas9 nuclease protein forms a complex with small guide RNAs, which are highly specific and complementary to target genomic DNA, enabling the implementation of high-throughput and multiplexed genome engineering strategies across various cell types and organisms (Jinek et al., 2012; Ran et al., 2013).

### 2.1 RNA interference

The discovery of RNA interference (RNAi) has profoundly transformed the field of gene silencing. Initially identified in *Caenorhabditis elegans*, RNAi has emerged as a powerful tool for combating viral and parasitic infections and for exploring gene functions (Fire et al., 1998; Kim and Rossi, 2008; Obbard et al., 2009; Rosso et al., 2009; Huvenne and Smagghe, 2010; Escobedo-Bonilla, 2013; Mohr and Perrimon, 2012). Despite its extensive applications, it is important to recognize that RNAi may result in hypomorphic phenotypes that do not fully recapitulate the effects of genetic mutations (Boettcher and McManus, 2015). Concerns regarding gene flow, horizontal gene transfer, and unintended effects on non-target organisms have motivated ongoing research to refine RNAi-based tools for safer and more effective gene manipulation.

RNAi is a highly conserved and potent mechanism for gene silencing, observed across various eukaryotic species, including plants, protozoa, nematodes, fungi, insects, and vertebrates. However, this process is absent in prokaryotes, highlighting its specialized role in eukaryotic cellular regulation (Das et al., 2011). RNAi acts as a defense mechanism against transposons and viruses, offering genome protection, particularly in plants (Obbard et al., 2009; Kemp et al., 2013; Nicolas et al., 2013).

The RNAi pathway is initiated by the introduction of double-stranded RNA (dsRNA) molecules that are perfectly complementary to the target gene. These dsRNAs are recognized and processed by the *RNase*III-like enzyme Dicer, generating small interfering RNAs (siRNAs) or microRNAs (miRNAs) (Gil-Humanes et al., 2010). These short RNAs, typically 20-24 base pairs long, possess 3′ hydroxyl termini, 2-nucleotide 3′ overhangs, and 5′ phosphorylated termini. They are incorporated into the RNA-induced silencing complex (RISC), where they guide sequence-specific degradation or translational repression of target messenger RNAs (mRNAs) (Das et al., 2011).

**Figure 1:**
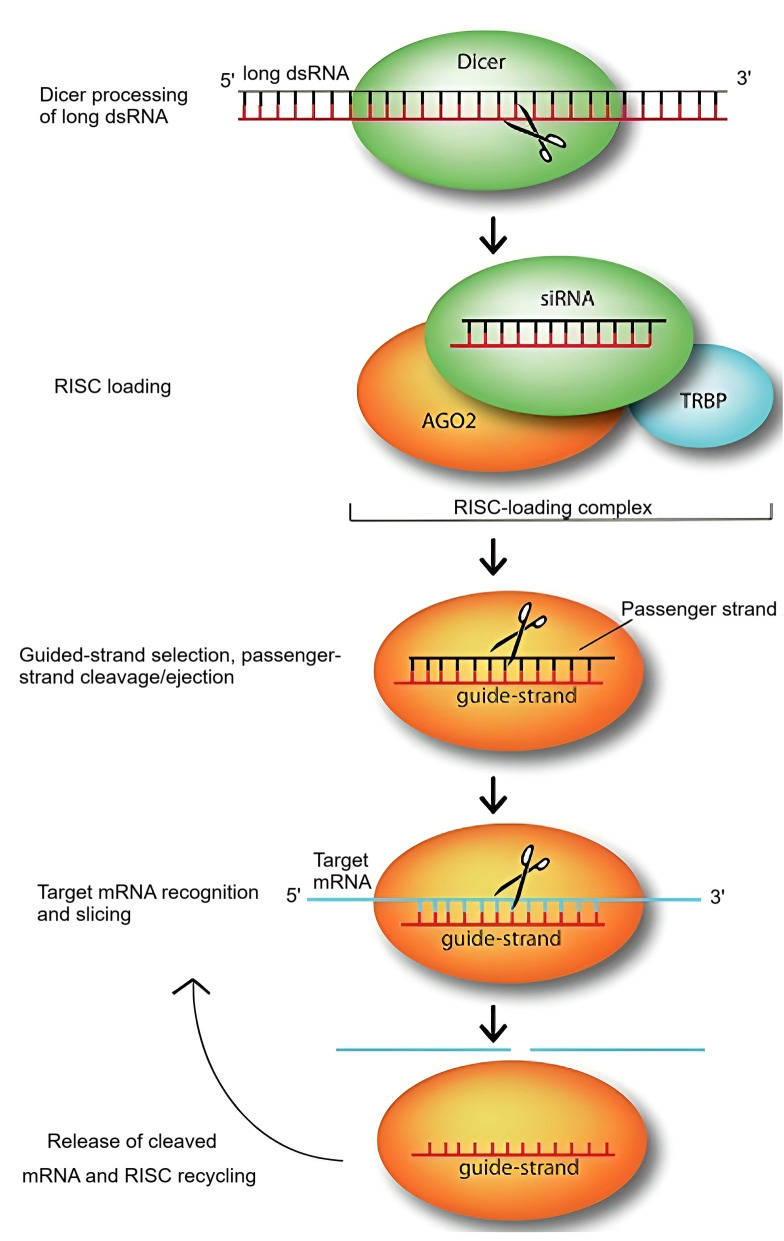
The RNA-mediated gene silencing pathway was first described by Jinek and Doudna in 2009. In this pathway, siRNA molecules play a crucial role in silencing target genes by guiding the sequence-dependent slicing of their target mRNAs. These non-coding RNAs initially exist as long dsRNA molecules, which are then processed by the endonuclease Dicer into short, active constructs of approximately 21-25 nucleotides. Once formed, a siRNA duplex is loaded onto Argonaute (AGO2), the central component of the RNA-induced silencing complex, with the assistance of the RNA-binding protein TRBP. AGO2 then selects the siRNA guide strand, cleaves, and removes the passenger strand. The guide strand, while bound to AGO2, pairs with its complementary target mRNA, allowing AGO2 to slice the target. After slicing, the cleaved target mRNA is released, and the RNA-induced silencing complex is recycled, utilizing the same guide strand for multiple rounds of slicing.

This process predominantly occurs in the cytoplasm. Additionally, signal amplification and intercellular propagation of RNAi have been observed, particularly in plants and *C. elegans*. The RNA-dependent RNA polymerase (RdRp) enzyme plays a pivotal role in amplifying siRNA signals and generating secondary dsRNAs, thereby enhancing the silencing effect (**Figure 1**) (Cohen and Xiong, 2011; Das et al., 2011).

Recent studies have demonstrated that RNAi can be triggered by various dsRNA molecules, including transposon transcripts, viral satellites, and transgenes (Barba and Hadidi, 2009). In plants, RNAi is more efficiently induced compared to mammals, nematodes, or flies. Instead of chemically synthesized short hairpin RNAs (shRNAs) or siRNAs, plants rely on expression cassettes that produce self-complementary hairpin RNAs (Hirai and Kodama, 2008; Eamens and Waterhouse, 2011). These cassettes are commonly incorporated into vectors such as pH/KANNIBAL and GATEWAY, which generate intron-containing hairpin RNAs (ihpRNAs) (Eamens and Waterhouse, 2011; Yan et al., 2012).

The transcription of RNAi-inducing cassettes in these vectors yields dsRNA molecules with two distinct regions: a single-stranded loop and a double-stranded stem (Eamens and Waterhouse, 2011). Following the development of RNAi vectors, advanced cloning strategies, including ligation-independent cloning (LIC), the Golden Gate cloning strategy, and one-step PCR cloning, have enabled efficient production of hairpin RNAs for gene silencing in plants, animals, and insects (Xu et al., 2010; Yan et al., 2012; Baghban-Kohnehrouz and Nayeri, 2016).

RNAi technology has been extensively employed to develop transgenic plants for improved traits, enhanced nutritional content, and better resistance to pests and diseases. For instance, RNAi has facilitated the production of seedless fruits, extended shelf life, modified flower colors, and improved secondary metabolite pathway. Moreover, RNAi-mediated genetic transformations have been instrumental in controlling plant pathogens and promoting sustainable agricultural practices (Das and Sherif, 2020). **Table 1** summarizes the applications of RNAi in various plant species for natural product biosynthesis.

**Table 1:** The latest advancements in genome editing for enhancing diverse traits and metabolites.

Plant	Method	Target locus	Trait improved	Ref
*Zea mays*	ZFN (DSBs)	ZmITPK	herbicide tolerance and the expected alteration of the inositol phosphate profile in developing seeds	Shukla et al., 2009
*Zea mays*	ZFN	Transgene with a “trait landing pad”	–	Ainley et al., 2013
*Nicotiana tabacum*	ZFN	ADH1	–	Townsend et al., 2009
*Nicotiana tabacum*	ZFN	*Disabled GUS*: *NPTII* transgene with a Zif268 target site	–	Wright et al., 2005
*Nicotiana tabacum*	ZFN	*CHN50*	–	Cai et al., 2009
*Nicotiana tabacum*	ZFN	*SurA* and *SurB*	–	Townsend et al., 2009
*Nicotiana tabacum*	ZFN	Transgene flanked with multiple CCR5-ZFN sites	–	Petolino et al., 2010
*Nicotiana tabacum*	ZFN	*mGUS* transgene with a QQR ZFN site	–	Marton et al., 2010
*Nicotiana tabacum*	ZFN	Transgene with two QQR ZFN sites	–	Weinthal et al., 2013
*Arabidopsis thaliana*	ZFN	gfp with hpt	recovery of hygromycin-resistant plants	Weinthal et al., 2013
*Arabidopsis thaliana*	ZFN	*At1g53430*, *At1g53440*, *At1g70450*, *At1g70460*, *At4g16960*, *At4g16940*, and *At4g1686*0	–	Qi et al., 2013
*Arabidopsis thaliana*	ZFN	*PPO*	–	de Pater et al., 2013
*Arabidopsis thaliana*	ZFN	*MPK8*, *MPK11*, *MKK9*, *MPK15 MAPKKK18*, and *GA3OX2*	–	Sander et al., 2010a
*Arabidopsis thaliana*	ZFN	*ABI4*	–	Osakabe et al., 2010
*Arabidopsis thaliana*	ZFN	*ADH1* and *TT4*	–	Zhang et al., 2010
*Petunia* sp.	ZFN	*mGUS* transgene with a QQR ZFN site	–	Marton et al., 2010
*Glycine max*	ZFN	*GFP* transgene; *DCL2a*, *DCL1b*, *DCL4a*, *DCL4b*, *RDR6a*, *RDR6b*, and *HEN1a*	–	Sander et al., 2010a; Curtin et al., 2011
*Glycine max*	ZFN	DCL2	–	Curtin et al., 2011
*Arabidopsis thaliana*	ZFN	ADH1	–	Qi and Zhang, 2014
*Triticum aestivum*	ZFN	GW2	–	Wang et al., 2014
*Triticum aestivum*	TALENs/ knock out	TaMLO homoeologs	develop wheat resistant to powdery mildew	Wang et al., 2014
*Solanum tuberosum*	TALENs/ knock out	VInv	reducing sugars during cold storage	Clasen et al., 2016
*Nicotiana tabacum*	TALENs	ALS gene	–	Zhang et al., 2013
*Triticum aestivum*	RNAi-mediated silencing	TaIPK1 TaABCC13	40–65 Reduction of phytic acid (%) 22–34 Reduction of phytic acid (%)	Aggarwal et al., 2018; Bhati et al., 2016
*Solanum lycopersicum*	RNAi-mediated silencing	Chalcone synthase 1 (CHS1)	Parthenocarpic fruit development	Schijlen et al., 2007
*Nicotiana tabacum*	RNAi-mediated silencing	*Flavanol synthase* (*FLS*)	Arrested seed set	Mahajan et al., 2011
*Malus × domestica*	RNAi-mediated silencing	Arabidopsis AGAMOUS	Seedless apple and colorless petals	Ireland et al., 2021
*Solanum lycopersicum*	RNAi-mediated silencing	*Pectate lyase* (*PL16*)	Fruit firmness, pericarp thickness, cell wall degradation, and shelf life	Ren et al., 2023
*Petunia × atkinsiana*	RNAi-mediated silencing	*Chalcone isomerase* (*CHI*)	Inhibited anthocyanin production	Keykha Akhar et al., 2023
*Cucumis sativus*	RNAi-mediated silencing	*Suc transporters* (*SUT1*)	Male sterile plants	Sun et al., 2019
*Camelina sativa*	RNAi-mediated silencing	*Fatty acyl-ACP thioesterase* (*FATB*)	Enhanced seed quality	Ozseyhan et al., 2018
*Triticum aestivum*	RNAi-mediated silencing	*Inositol pentakisphosphate kinase* (*IPK1*)	Enhanced micronutrient	Aggarwal et al., 2018
*Solanum melongena* L.	RNAi-mediated silencing	*Constitutive photomorphogenic 1* (*COP1*)	Improved anthocyanin, fruit size, and chlorogenic acid reduced	Li et al., 2023
*Carica papaya* L.	RNAi-mediated silencing	*DE-ETIOLATED* (*DET1*)	Secondary metabolite pathway modification	Jamaluddin et al., 2019
*Triticum aestivum*	meganuclease	*DsRed reporter*	–	Youssef et al., 2018
*Zea mays*	meganuclease	*BAR*	confer resistance to phosphinothricin	D’Halluin et al., 2008
*Oryza sativa*	CRISPR/Cas9	B-type response regulator (RR22)	Enhanced salt tolerance	Zhang et al., 2019
*Oryza sativa*	CRISPR/Cas9	Basic helix-loop-helix 024 (bHLH024)	Enhanced salt tolerance	Alam et al., 2022
*Oryza sativa*	CRISPR/Cas9	*Drought and salt tolerance* (*DST*)	Enhanced salt tolerance and drought tolerance	Santosh Kumar et al., 2020
*Oryza sativa*	CRISPR/Cas9	REC8, PAIR1, OSD1, BBM1	Clonal seeds production	Khanday et al., 2019
*Oryza sativa*	CRISPR/Cas9	*REC8, PAIR1, OSD1, BBM1 in single step*	Synthetic apomixis in F1 hybrid	Vernet et al., 2022
*Oryza sativa*	CRISPR/Cas9	*PAIR1, REC8, OSD1, BBM4*	Synthetic apomixis	Wei et al., 2023
*Oryza sativa*	CRISPR/Cas9	*SPO11-1, REC8, OSD1, MATL*	Apomixis	Xie et al., 2019
*Oryza sativa*	CRISPR/Cas9	*ONAC127, ONAC129*	Decreased seed set under heat stress	Ren et al., 2021
*Oryza sativa*	CRISPR/Cas system	*OsOr gene*	enhance the formation of b-carotene in the crop of rice endosperm	Endo et al., 2019
*Oryza sativa*	CRISPR/Cas system	*OsF3′H, OsDFR, OsLDOX* *OsTTG1*	the process of anthocyanin biosynthesis	Jung et al., 2019; Yang et al., 2021
*Oryza sativa*	CRISPR/Cas9	*OsGBSSI*	Low amylose content	Xu et al., 2021
*Oryza sativa*	CRISPR/Cas9	*ENO* *CLV3* *GS3, Gn1a* *GW2, GW5, TGW6* *GL2/OsGRF4, OsGRF3* *GS9* *GW5* *OsGS3, OsGW2 and OsGn1a* *Gn1a, GS3, DEP1*	Fruit size Fruit size Grain length Grain length and width Grain size Slender grain shape Grain width Grain length and width Larger grain size, enhanced grain number, and dense erect panicles	Yuste-Lisbona et al., 2020 Zsögön et al., 2018 Shen et al., 2018 Xu et al., 2016 Hao et al., 2019 Zhao et al., 2018 Liu et al., 2017 Zhou et al., 2019 Li et al., 2016
*Triticum aestivum*	CRISPR/Cas9	*TaGW2, TaARR12*	Increased drought resistance and grain yield	Li et al., 2024
*Tritocum aestivum*	CRISPR/Cas9	*EDR1*	Developing resistance to powdery mildew disease caused by *Blumeria graminis* f.sp. (Btg) Tricitici	Zhang et al., 2017
*Zea mays*	CRISPR/Cas9	*ARGOS8*	Increased grain yield drought stress	Shi et al., 2017
*Camellia sinensis*	CRISPR/Cas	*CsHB1*	reduction in caffeine accumulation	Ma et al., 2021
*Glycine max*	CRISPR/Cas9	GmIPK1	reduction in PA content in soybean T2 seeds	Song et al., 2022
*Solanum lycopersicum*	CRISPR/Cas9	*ALS*	Resistance to bacteria speck disease	Ortigosa et al., 2019
*Solanum lycopersicum*	CRISPR/Cas9	*ANT1*	Fruit color (purple)	Vu et al., 2020
*Solanum lycopersicum*	CRISPR/Cas9	*SlIAA9*	Parthenocarpy	Ueta et al., 2017; Nguyen et al., 2024
*Solanum lycopersicum*	CRISPR/Cas9	*SlAGL6*	Parthenocarpy	Gupta et al., 2021
*Solanum lycopersicum*	CRISPR/Cas9	*SPO11-1, REC8, TAM, OSD1*	Clonal gamete production	Wang et al., 2024
*Solanum lycopersicum*	CRISPR/Cas9	*GID1α*	Higher harvest index under drought stress	Illouz-Eliaz et al., 2020
*Solanum lycopersicum*	CRISPR/Cas9	*SlGT30*	Enhanced drought resistance, increased fruit size and weight	Lv et al., 2024
*Zea mays*	CRISPR/Cas9	*ALS*	Herbicide resistance	Svitashev et al., 2015
*Citrus paradisi*	CRISPR/Cas9	*CsLOB1 promoter*	Resistance to citrus canker disease	Jia et al., 2017
*Solanum tuberosum*	CRISPR/Cas9	*elF4E and elF(iso)4E)*	Resistance against viruses and cold-induced sweetening	Hameed et al., 2020
*Glycine max*	CRISPR/Cas9	*HaHB4*	Drought tolerance	Martignago et al., 2020
*Musa paradisiaca*	CRISPR/Cas9	*MaACO1*	Long shelf life	Hu et al., 2021
*Physalis* sp.	CRISPR/Cas9	*ClV1*	Fruit size	Lemmon et al., 2018

### 2.2 Meganucleases

Recognizing the significance of DSBs in genome engineering, the initial artificial system to generate site-specific DSBs involved the use of meganucleases (homing endonucleases). This approach triggered DNA repair pathways, resulting in specific modifications to the DNA, such as indels or single nucleotide polymorphisms (SNPs) in eukaryotic genomes. One of the first meganucleases to be discovered and characterized was the I-SceI meganuclease, which was identified in the 1970s and 1980s from the mitochondria of *S. cerevisiae* (yeast) (Jacquier and Dujon, 1985; Bos et al., 1978; Faye et al., 1979).

A rare-cutting enzyme, produced via an intron within the mitochondrial large 21 S ribosomal RNA subunit (LsrRNA), selectively identifies and cuts an 18 bp sequence in an intron-less version of the LsrRNA gene, causing a double-strand break (DSB) (Jacquier and Dujon, 1985; Stoddard, 2014). The homologous recombination (HR) pathway rectifies the resultant double-strand breaks (DSBs) by employing a variant of the 21 S rRNA gene that contains an intron, thereby facilitating the incorporation of the intron-embedded I*-*SceI open reading frame (ORF) region into the designated target locus (Jacquier and Dujon, 1985). The I*-*SceI endonuclease, characterized by its extensive DNA recognition sequence and pronounced specificity, can be expressed in a manner that does not compromise the integrity of host genomes or cellular structures. As a result, I*-*SceI has been effectively harnessed to induce DSBs at targeted loci, allowing for the investigation of DNA repair mechanisms across various eukaryotic genomes (Rouet et al., 1994a, 1994b; Choulika et al., 1995). In the year 1993, Puchta et al., demonstrated that the DSBs generated by I-SceI enhance HR not only in plant systems but also in *Nicotiana tabacum* (Puchta et al., 1993; Puchta, 1999).

Meganucleases require the ability to design nucleases with a high level of sequence specificity to achieve exact genome modification. However, one of the challenges in engineering these nucleases is the overlap between the DNA-binding domains and cleavage (Stoddard, 2011). This overlap can hinder the design process. On the other hand, altering the amino acid sequence to gain new DNA sequence particularity often compromises the catalytic action of the enzyme. To overcome this challenge, a semi-rational approach has been developed. This approach involves using the mathematical examination of the structural aspects of the protein-DNA interface to generate meganucleases with new specificities. This strategy has shown promise in the field (Ashworth et al., 2006).

The two-step combinatorial method has made it easier to create personalized meganucleases through a wider span of target sites. This has expanded the usefulness of designer meganucleases for different purposes. Among these is I-CreI, which serves as a molecular framework for generating innovative meganucleases with unique specificities. Nevertheless, there are instances where their binding and/or cleavage characteristics may not be optimal. But, by optimizing the framework, we can improve both the nuclease specificity and activity. Different approaches have been explored to achieve this goal (Arnould et al., 2007; Redondo et al., 2008; Grizot et al., 2009).

Meganucleases were assessed for their potential application in tomato and oilseed rape crops. Both I-SceI and a tailored meganuclease have been identified as capable of inducing homology-directed repair (HDR) and double-strand breaks mediated recombination in a reporter gene (Rahmanian and Seyeddokht, 2023). Despite being less effective than I-SceI, the customized meganuclease was successful in causing the removal of an exogenous transgene in tomato plants (Danilo et al., 2022). Engineered meganucleases based on I*-Crel* have been created to exhibit enhanced activity in recognizing sequences with specific central sequences. The development of the ARCUS platform represents a new generation of meganuclease technology that enables the production of nucleases with tailored activity and specificity, including the ability to differentiate between target sites that vary by just 1 base pair (Bartsevich et al., 2016).

Youssef et al. (2018) described for the first time the use of a customized meganuclease to cleave wheat DNA *in vivo*. They showed that double excisions removed previously inserted DNA cassettes containing the DsRed reporter gene and, in many cases, the meganuclease target site was correctly reconstructed, providing opportunities for subsequent insertion of accumulated genes to replace the selected gene. I-SceI nuclease catalyzed the precise integration of gene in maize at a pre-integrated target site, thereby inducing expression of the BAR gene (the BAR gene is known to confer resistance to phosphinothricin) (D’Halluin et al., 2008).

Studies have shown that the effectiveness of meganucleases can be impacted by various factors related to the chromosome context of the target sequences (Kim et al., 1996; Smith et al., 2000; Durai et al., 2005). However, the low frequency of genome editing in somatic cells was one of the major limitations of I*-*SceI and other meganucleases, recognition of natural target sites in the genome could not be easily available (Porteus, 2015). Therefore, to solve this problem, ZFNs and TALENs technologies with endonuclease activity domains for inducing targeted DSBs at specific genomic DNA loci have been developed.

### 2.3 Zinc finger nucleases

Zinc finger nucleases (ZFNs) are engineered nucleases designed to create precise DSBs in DNA at specific genomic locations (Memon, 2021). These breaks can trigger DNA repair mechanisms, resulting in targeted mutagenesis or chromosomal segment removal via non-homologous end joining (NHEJ) or gene targeting through homologous recombination (HR) (Van Eck, 2020). The discovery of zinc finger protein domains in the early 1990s revolutionized genome engineering in model plants and crops. Using ZFNs to target specific sequences in genomic DNA enables various modifications through DNA repair pathways like NHEJ and HR (Weinthal et al., 2010; Tzfira et al., 2012; Voytas, 2013).

**Figure 2:**
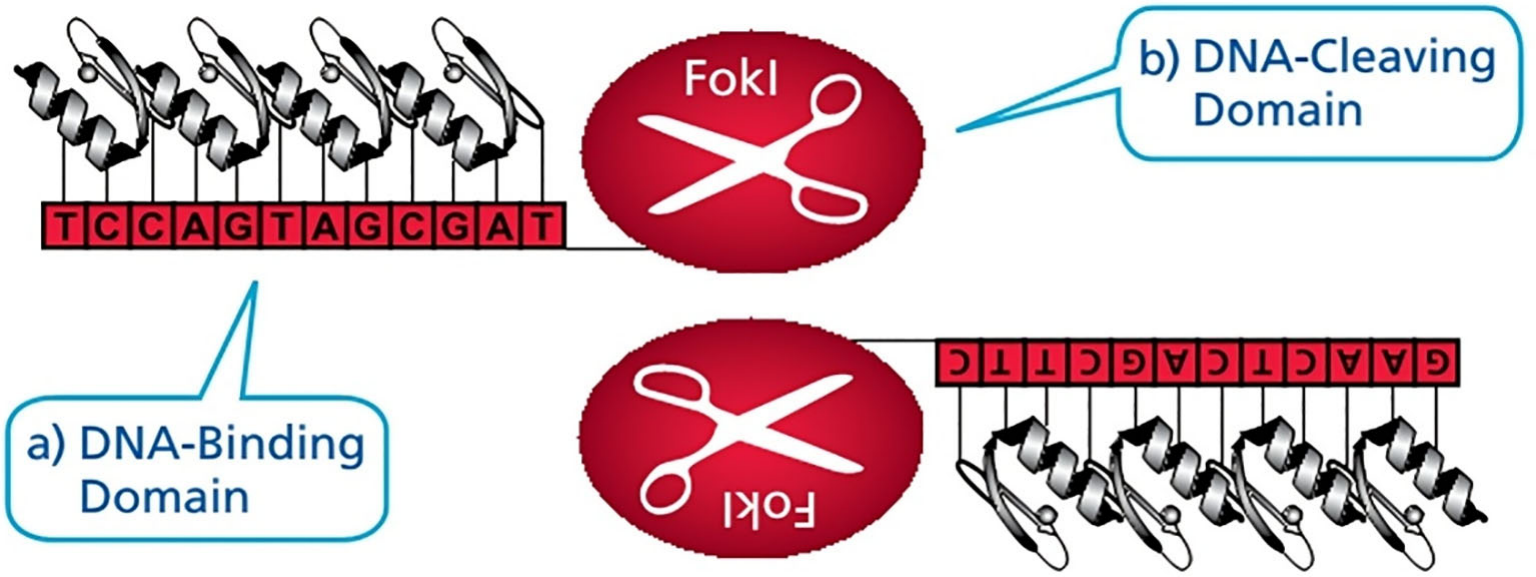
Zinc finger nucleases: as highly-specific ‘genomic scissors’ (Simmons and Douglas, 2016).

ZFNs are vital in activating nuclease dimers that cleave DNA away from the binding site. Each monomer contains a zinc finger DNA-binding domain-spanning 30 amino acids and featuring arrays of Cys2-His2 fingers with a Zn^2+^ binding ion—and a nonspecific *FokI* nuclease domain (**Figure 2**). The zinc finger domain typically includes three or four patterns, with each pattern recognizing a specific group of three DNA bases. This enables precise recognition of an 18 or 24-base pair sequence using a ZFN pair (Zhang et al., 2015; Simmons and Douglas, 2016).

Advances in ZFN engineering have been achieved through two major platforms. The first, modular assembly, combines individual fingers with specific DNA-binding properties (Beerli and Barbas, 2002; Liu et al., 2002; Bae et al., 2003; Segal et al., 2003; Mandell and Barbas, 2006). However, modular assembly often has low efficiency (≤30%) and may exhibit limited activity or high toxicity (Cornu et al., 2008; Ramirez et al., 2008; Kim et al., 2011). The second approach, based on screening multi-finger databases, considers interactions between neighboring fingers. Sangamo Bioscience pioneered this method, branding it as CompoZr^®^ from Sigma Aldrich^®^ (Doyon et al., 2008). Academic methods include oligomerized pool engineering (OPEN) (Maeder et al., 2008) and context-dependent assembly (CoDA) (Sander et al., 2010a), both using improved two-finger databases (Gupta et al., 2012).

Online tools such as ZiFiT Targeter Version 4.2 (http://zifit.partners.org/ZiFiT/) (Sander et al., 2007, 2010b), Zinc Finger Database (ZiFDB 2.0) (https://zifdb.msi.umn.edu:8444/ZiFDB), and ZFNGenome (Reyon et al., 2011) facilitate ZFN design and selection through modular synthesis. Researchers continue exploring ZFN-mediated genome modifications like targeted mutagenesis, gene replacement, and stacking via HR repair pathways in various crops (**Table 1**).

Despite its potential, ZFN technology faces challenges, including germinal transmission, chromatin accessibility, and off-target effects. ZFN-induced DSBs often result in small deletions (≤50 bp). Achieving large chromosomal deletions requires simultaneous creation of DSBs at both ends, with deletion frequencies remaining low (Qi et al., 2013). Tools have been developed to predict off-target sites in genomes outside plants (Cradick et al., 2011). In Arabidopsis, potential off-targets are identified using BLAST scans and verified for ZFN activity (Zhang et al., 2010). High-throughput methods have assessed off-target effects in maize. However, a major drawback of ZFNs remains the high cost and complexity of designing high-affinity DNA-binding domains essential for genome editing (Shukla et al., 2009).

ZFN-mediated genome modifications have been achieved in various plants, including Arabidopsis, petunia, tobacco, soybean, and maize (Zala et al., 2016). Although ZFN-induced modifications are often successful in somatic cells, germ cell modification rates remain low (Kamburova et al., 2017). Challenges such as low germ cell transmission and cellular toxicity must be addressed to maximize ZFN potential in plant genetic engineering (Qi, 2015).

The applications of ZFNs in targeting specific genes in plants include various examples. For instance, the *ADH1* gene in tobacco, encoding the enzyme alcohol dehydrogenase, was targeted to investigate the feasibility of site-specific mutagenesis in plants and served as a model system for functional genomics (Townsend et al., 2009). Similarly, in Arabidopsis, targeted deletions and insertions in the *ADH1* gene demonstrated the precision of ZFNs in genome editing (Qi and Zhang, 2014). In soybean, the *DCL2* gene, involved in virus-induced gene silencing, was knocked out to enhance resistance to viral pathogens (Curtin et al., 2011). Additionally, in wheat, the *GW2* gene, associated with grain weight, was edited to improve crop yield by enhancing grain size and weight (Wang et al., 2014).

### 2.4 Transcription activator-like effector nucleases

**Figure 3:**
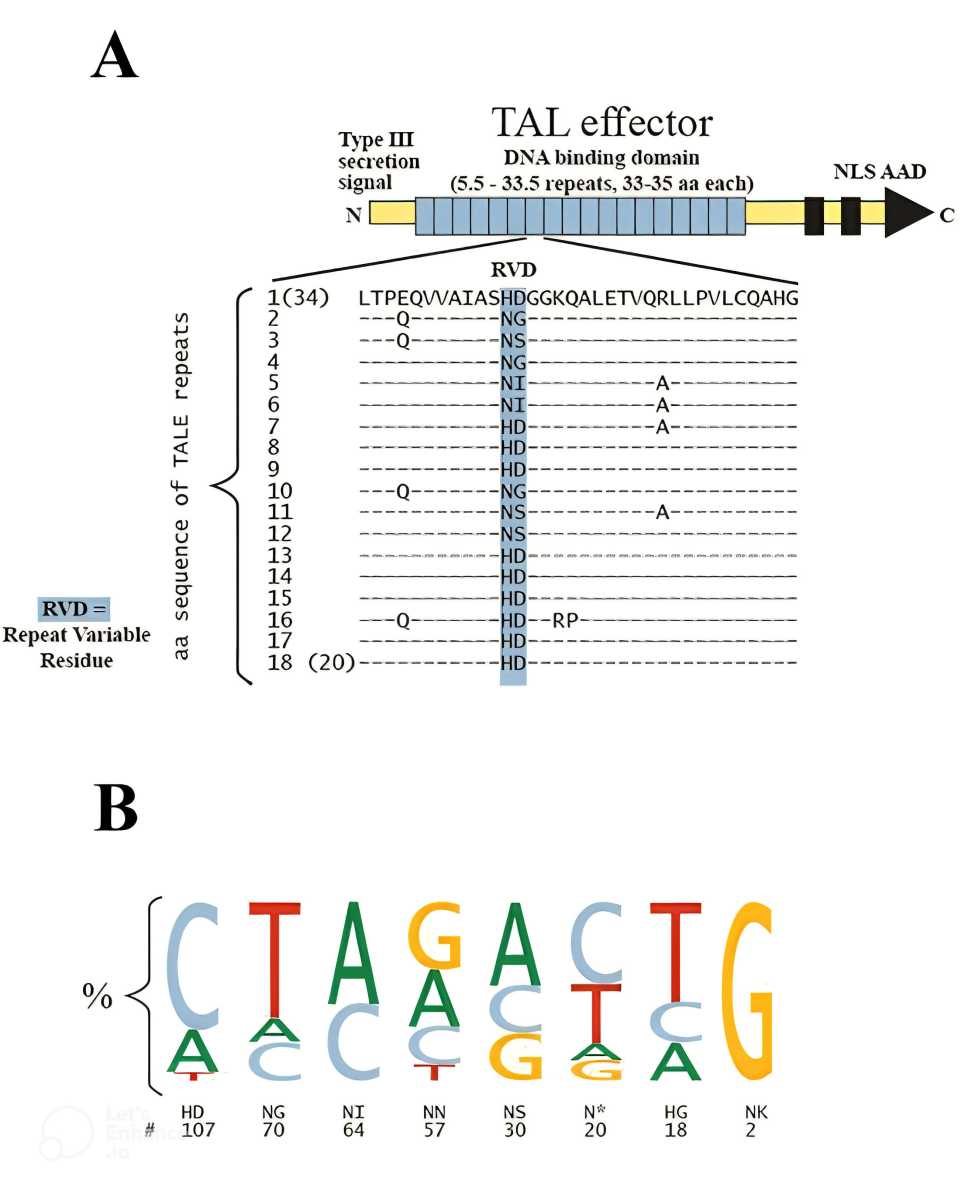
Diagram of a TAL effector structure and sequence. **(A)** TALE protein visualization. The middle section of repeats (represented by blue squares) is responsible for DNA binding. Additionally, the nuclear localization signals (NLS) and acidic activation domains (AAD) are included in the illustration. The amino acid sequences for individual repeats within a typical array are displayed; repeat variable di-residues (RVDs) are highlighted in blue, while dashes indicate conserved amino acid residues. **(B)** Frequencies of RVD-nucleotide associations. The size of the letter in the sequence logo indicates how often RVDs are linked to specific bases (Moscou and Bogdanove, 2009).

Transcription Activator-Like Effector Nucleases (TALENs) are a highly effective gene-editing technology, capable of precisely modifying specific genomic sequences. TALENs consist of a DNA-binding domain that interacts with the target DNA and is connected to a DNA cleavage domain, enabling the induction DSBs at specific locations in the genome. These DSBs can be repaired through either homology-directed repair (HDR) or error-prone nonhomologous end joining (NHEJ), thereby significantly enhancing transformation efficiency (Harale et al., 2022). Initially discovered in *Xanthomonas species*, TALEs are a group of DNA-binding nuclease proteins that infect a variety of plants, including citrus, rice, pepper, cotton, and soybean (Mansfield et al., 2012). Research on TALEs has revealed their ability to bind to genomic DNA and activate gene expression by mimicking eukaryotic transcription factors (ETFs) (Nemudryi et al., 2014). The structure of TAL effector proteins includes an N-terminal type III secretion domain, a central DNA-binding region known as the repeat array, which consists of several repeats (ranging from 1.5 to 33.5 repeats of 34 amino acids each), and a C-terminal region that contains multiple nuclear localization signals (NLS) and an acidic activation domain (AAD), characteristic features of prokaryotic transcription factors (TF) (**Figure 3A**) (Boch and Bonas, 2010). Notably, there is significant variability in the sequence of TAL effector proteins, particularly at the C- and N-termini, as well as within the repeat array. The 12th and 13th residues within each repeat, known as repeat-variable di-residues (RVDs), display the greatest variability. While over 20 different RVDs have been identified in natural TAL effectors, four RVDs including HD (binding to C), NI (paired with A), NG (linked to T), and NN (recognizing both G and A) are the most prevalent, accounting for over 75% of RVDs (**Figure 3B**) (Moscou and Bogdanove, 2009). A conserved T at the -1 position is a common feature in all TAL effector sequences; any alteration to this T results in a decrease in gene upregulation (Boch et al., 2009).

**Figure 4:**
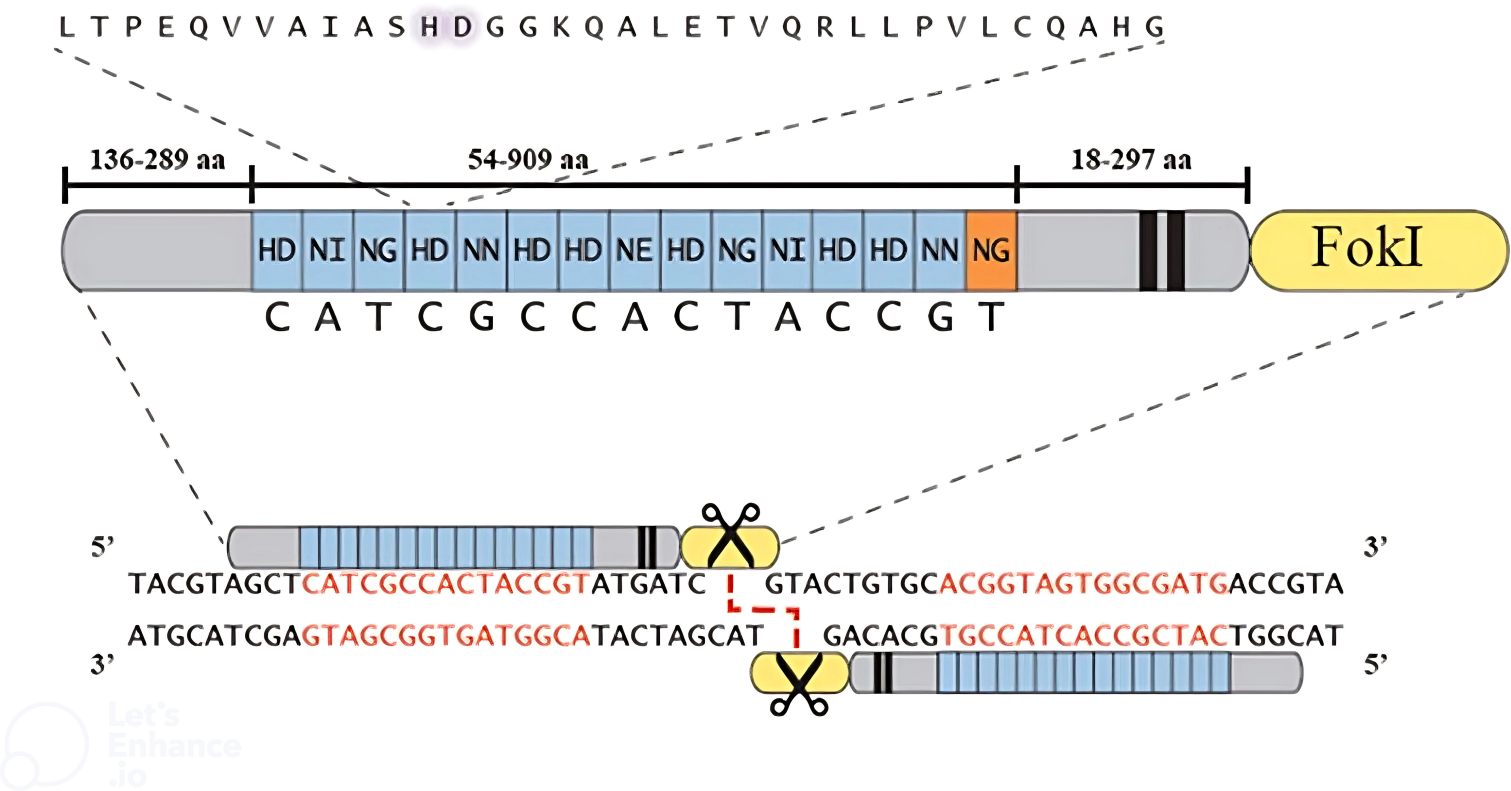
A depiction of the TALEN structure. The extended perspective of the TALEN displays the sizes of different sections, including a typical TAL effector array and its corresponding nucleotide target. The alignment of a complete TALEN pair is illustrated, with the TALEN target sequence emphasized in red (Christian and Voytas, 2015).

TAL effector nucleases (TALENs) are engineered to function as dimers, with each monomer containing the catalytic domain of *FokI* nuclease. These TALENs have shown superior efficiency compared to zinc finger nucleases (ZFNs) in generating high-throughput DSBs in the target DNA (Christian et al., 2010). TALEN monomers are designed to bind to two half-sites of DNA with a spacer sequence between them. This unique design allows *FokI* monomers to dimerize and create a double-strand break in the spacer sequence separating the half-sites (**Figure 4**) (Christian et al., 2010). Initially, designing new TAL effector arrays to recognize a specific sequence was time-consuming; however, researchers have developed efficient strategies to address this challenge. Numerous online tools are now available for designing and selecting TALEN pairs, including TAL Effector Nucleotide Targeter 2.0 (TALE-NT), Scoring Algorithm for Predicting TALEN Activity (SAPTA), TAL Effectors, E-TALEN, CHOPCHOP, TALEN Designer, ZiFit, and Mojo Hand (Gaj et al., 2013).

A widely adopted method for producing high-throughput engineered TALENs is the Golden Gate cloning system. This system facilitates the sequential assembly of multiple DNA fragments in a single reaction using Type IIS restriction endonucleases (Engler et al., 2008, 2009; Briggs et al., 2012; Reyon et al., 2012; Schmid-Burgk et al., 2013). The application of the Golden Gate method has greatly accelerated the development of novel TAL effector arrays and has further advanced genome engineering. TALENs have diverse applications, including enhancing plant traits through the introduction of novel genes and regulating gene expression (Behboudi et al., 2022). Additionally, TALENs have been employed to generate OVM-knockout chicken eggs, offering a potential source of safe, allergen-free food (Ezaki et al., 2023). In pigs, TALENs have been used to produce genetically inheritable knockout pigs with a mutation rate comparable to wild-type controls, making them a reliable resource for clinical applications (Barnett, 2018). The precision and safety of TALEN-based genome editing make them invaluable tools in genetic engineering (Choi et al., 2020).

Over the years, numerous studies have documented TALEN-mediated genome modifications in approximately 17 different model organisms, including a variety of plant species such as Arabidopsis protoplasts, tobacco, barley, rice, and Brachypodium (Cermak et al., 2011; Li et al., 2012; Mahfouz et al., 2012; Shan et al., 2013a; Wendt et al., 2013; Zhang et al., 2013). These studies highlight the potential of TALENs to revolutionize agricultural practices by enabling targeted genome editing in crops and livestock. For instance, TALENs have been used to knock out three *TaMLO* homoeologs in wheat, conferring resistance to powdery mildew (Wang et al., 2014). They have also been employed to generate rice resistant to *Xanthomonas oryzae* pv. *oryzae* (Li et al., 2012) and to knockout the vacuolar invertase (*VInv*) gene in potatoes, resulting in tubers with negligible levels of harmful reducing sugars during cold storage (Clasen et al., 2016). Furthermore, Zhang et al. (2013) described targeted genome modification in tobacco (*Nicotiana tabacum*) protoplasts, with TALENs directed at the ALS gene. Despite these advances, the construction of TALENs requires the development and synthesis of a new nuclease for each target DNA sequence, as well as the re-engineering of TALEN or ZFN. Therefore, assembling TALENs is a time-consuming and costly process that requires significant expertise in experimental design and molecular biology (Gaj et al., 2013).

### 2.5 CRISPR

#### 2.5.1 CRISPR/Cas9

**Figure 5:**
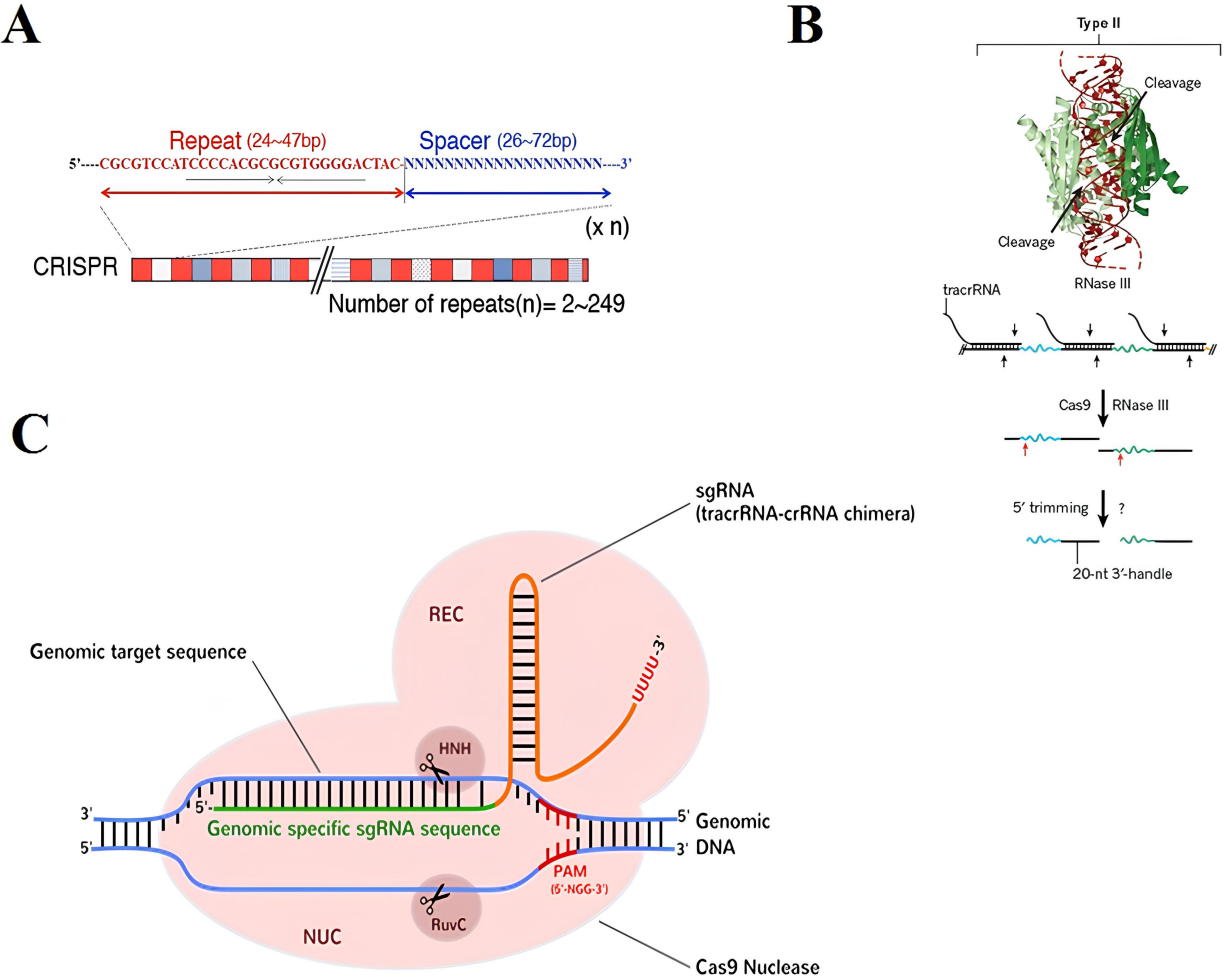
**(A)** CRISPR loci are composed of approximately 24-47 bp palindromic repeat sequences (highlighted in red), which are interspersed with 26-72 bp spacer sequences (highlighted in blue). These spacer sequences do not share any common features in terms of their sequences. The maximum number of repeats can reach up to 249, as reported by Kim and Kim, 2014. **(B)** In type II CRISPR systems, tracrRNA binds to the pre-crRNA repeat to create duplex RNAs that are then cleaved by the host RNase III (PDB ID: 2EZ6), a process that may also involve Cas9. **(C)** Subsequent trimming of the leftover repeat sequences from the 5ʹ end is carried out by an unidentified nuclease, as described by Wiedenheft et al., 2012. The activation of Cas9 protein occurs through the binding of gRNA. This binding induces a conformational change in the Cas9 protein, leading to the activation of its nuclease activity. The RuvC and HNH domains are responsible for the specific and efficient cleavage of the target DNA when complemented with crRNA (highlighted in green), (Jinek et al., 2014).

The CRISPR/Cas system, which serves as an acquired immune system protecting bacterial cells against invading bacteriophages and DNA plasmids, was initially discovered in the genome of *Escherichia coli* (Ishino et al., 1987). CRISPR loci have been found in both Archaea and bacterial genomes, with relevance rates of 86% and 45%, respectively, as reported by CRISPRdb (Grissa et al., 2007). These loci typically consist of two main components: a group of CRISPR-associated (*Cas*) genes, such as *Cas1-4*, which are crucial for triggering a bacterial immune response against bacteriophages, and the CRISPR arrays, which are composed of repeated sequences (25-50 bp) that number more than 249 and are separated by variable sequences (spacers) that match sequences found in foreign genetic elements (protospacers), which are 26-72 bp long (Kim and Kim, 2014) (**Figure 5A**). *Cas* genes are transcribed into proteins, while most CRISPR arrays are initially transcribed as a single RNA, which is subsequently processed into shorter CRISPR RNAs (crRNAs). These crRNAs guide specific Cas enzymes to target and degrade nucleic acids in the genomic DNA. Additionally, the leader sequence, typically 200-500 bp long and containing AT-rich sequences, acts as a promoter for the CRISPR loci. The natural mechanism of microbial CRISPR systems in adaptive immunity involves three main steps: (1) phage infection, where genetic elements from bacteriophages or plasmids invade the cell; (2) spacer acquisition, where specific CRISPR-associated (Cas) enzymes acquire spacers from external protospacer sequences and integrate them into the CRISPR locus in the prokaryotic genome; and (3) crRNA biogenesis and processing, where these spacers are separated by repeated sequences, enabling the CRISPR system to distinguish between self and non-self. Three categories of CRISPR/Cas systems have been identified based on core components and sequences (Makarova et al., 2011; Wiedenheft et al., 2012; Barrangou, 2013; Chen and Gao, 2014). The type I and type III systems require the formation of a large functional multi-Cas complex, while the type II system relies on a single Cas9 protein. In type II CRISPR, the direct repeats are paired with a trans-activating CRISPR RNA (tracrRNA) to form an RNA duplex. This duplex is cleaved and processed by endogenous RNase III and other nucleases. The crRNA-tracrRNA hybrids then interact with Cas9 to interfere with and degrade the target DNA sequence. The target DNA sequence is fully paired with the dual guide RNA (gRNA) consisting of a crRNA-tracrRNA hybrid (**Figure 5B**) (Hsu et al., 2014).

Precisely targeting a specific DNA sequence is essential for genome editing in an organism. In the CRISPR/Cas9 system, the Cas9 protein and guide RNA work together to identify target sequences with high accuracy. The Cas9 protein consists of six domains, with the REC I domain binding to the guide RNA, while the function of the REC II domain is still under investigation. The arginine-rich bridge helix triggers cleavage upon target DNA binding, while the PAM-interacting domain ensures specificity in binding (Nishimasu et al., 2014; Anders et al., 2014; Jinek et al., 2014). Additionally, the HNH and RuvC domains function as nuclease domains capable of cutting single-stranded DNA. These domains share significant similarities with the HNH and RuvC domains found in other proteins (Jinek et al., 2014; Nishimasu et al., 2014). Without the guide RNA (gRNA), which consists of crRNA that perfectly matches the target DNA sequence and tracrRNA that forms a T-shaped structure with one tetraloop and two or three stem loops, the Cas9 protein remains inactive (Jinek et al., 2012, 2014; Nishimasu et al., 2014). Upon binding of the gRNA to the Cas9 protein complex, the interaction between the protein side chains and RNA bases induces changes in the protein’s structure, transitioning it from inactive to active (Jinek et al., 2014). After the formation of the ribonucleoprotein complex (Barrangou, 2013), the targeted Cas9 cuts the protospacer DNA using the HNH nuclease and RuvC-like domains. These domains cut the complementary and non-complementary strands of the DNA, respectively. The precise cleavage occurs three base pairs before the protospacer adjacent motif (PAM) sequence, typically represented by the 5’-NGG-3’ sequence from *Streptococcus pyogenes*, resulting in a blunt end (**Figure 5C**). The specificity of the gRNA is determined by the seed region, which is about 12 bases before the PAM sequence and must match between the gRNA and the target dsDNA. The gRNA-Cas9 complex facilitates genome editing by creating a double-stranded break (DSB) at the target genomic locus, and the repair of this break usually occurs through either the NHEJ or HDR pathways for DNA damage repair (Deltcheva et al., 2011; Barrangou, 2013).

CRISPR/Cas9 technology faces a significant challenge due to the relatively high occurrence of off-target mutations, as indicated by previous studies (Cong et al., 2013; Jiang et al., 2013; Mali et al., 2013; Pattanayak et al., 2013). To enhance gRNA targeting efficiency, a 20 nt sequence specificity in the gRNA is crucial. However, research findings suggest that the 8-12 nt at the 3′-end (seed region) play a vital role in accurate target site recognition and cleavage (Cong et al., 2013; Jinek et al., 2012). Nonetheless, the tolerance for multiple mismatches in the PAM-distal region depends on the number and arrangement of the mismatches (Fu et al., 2013; Hsu et al., 2014). One advantage of the CRISPR/Cas9 system is its reprogrammability, which allows for the rapid and cost-effective examination of gRNAs for off-target effects. Unlike ZFNs and TALENs, the *FokI* nuclease domain of Cas9 functions as a dimer, with each catalytic monomer (nickase) cleaving a single DNA strand to produce a staggered DSB with overhangs. A mutated form of Cas9, with a D10A mutation in the RuvC nuclease domain, has been developed to convert it into a nickase. Consequently, the use of two Cas9 nickases as a dimer can lead to the creation of sticky-ended DSBs, thereby improving Cas9 specificity for target cleavage. This results in a significant increase in genome modification specificity in human and mouse cells, ranging from 50 to 1500-fold (Mali et al., 2013; Ran et al., 2013; Cho et al., 2014), as well as in Arabidopsis (Fauser et al., 2014; Schiml et al., 2014). To overcome the challenges associated with the requirement for two equally efficient gRNAs and a catalytically active paired nickase system, scientists have developed hybrids of catalytically inactive Cas9 and *FokI* nuclease. These hybrids have shown similar efficacy to the nickases, but with significantly enhanced specificity (up to 140-fold) compared to the wild-type enzyme (Cho et al., 2014; Guilinger et al., 2014; Tsai et al., 2014).

CRISPR-mediated genome modification is quickly emerging as a potent technique for genome engineering due to its various features and capabilities. This innovative technology has the potential to be utilized for quick and effective genome editing, as well as genome regulation, in a diverse array of applications. Additionally, Cas9 nuclease stands out for its ability to deliver precise and effective genome modifications in comparison to alternative genetic engineering techniques across many plant species. These applications can be implemented through: 1) gene disruption via the NHEJ repair pathway (also referred to as without donor template DNA), 2) gene knock-out utilizing the HDR repair pathway (with a reporter knock-in), 3) disruption of non-protein coding genes, 4) introducing specific mutations, including: a) desired SNPs introduction or correction, b) desired insertion or deletion, c) tagging the endogenous genes using genetically encoded molecular tags in the context of functional analysis, protein purification, or investigations into protein and RNA localization, 5) promoter analysis, 6) conditional knockout for essential genes or tissue-specific research by inserting LoxP sites around the exon to be knocked out, 7) creating large chromosomal deletions using two sgRNAs to induce DSBs at sites flanking the region of interest, 8) CRISPR interference (CRISPRi) resulting in transcriptional reduction of the target RNA, akin to TALE transcriptional repression (Gilbert et al., 2013), and 9) CRISPR activation (CRISPRa) for achieving maximal activation (Gilbert et al., 2014; Konermann et al., 2015).

In plant biology, the continuous expression of Cas9 has been achieved using various promoters, such as *35sCaMV*, *OsAct1*, *35SPPDK*, or *UBQ* (Nekrasov et al., 2013; Shan et al., 2013b; Xie and Yang, 2013; Jiang et al., 2013; Li et al., 2013; Mao et al., 2013). Similarly, the sgRNA, a crucial component of the CRISPR system, is frequently modified to increase its expression using U3 or U6 RNA polymerase III promoters from species like Arabidopsis, rice, or wheat. The configuration of sgRNA depends on the target sequence, with a commonly used 20 bp target sequence adjacent to the PAM sequence (N)20NGG in mammalian genome editing, and (N)19-20NGG sequences in plants (Miao et al., 2013; Shan et al., 2013b; Feng et al., 2014). Notably, studies in plant science have shown that the sgRNA does not always require an exact match. For instance, the U6 promoter in G(N)19-20 or the U3 promoter in A(N)19-20 does not need the “G” or “A” for determining the transcription start site (Shan et al., 2013b). Many online tools are available for designing effective and precise sgRNA molecules for targeting DNA sequences, as summarized in **Table 2**.

**Table 2:** Web server sources to design gRNA in CRISPR/Cas system (adopted from Khatodia et al. 2016).

Software	Description	Source
Addgene	Materials and resources	https://www.addgene.org/crispr
sgRNA Designer	gRNA design tool	http://broadinstitute.org/rnai/public/analysis-tools/sgrna-design
Cas9 Design	gRNA design tool	http://cas9.cbi.pku.edu.cn
CHOPCHOP	Target DNA identification tool	https://chopchop.rc.fas.harvard.edu
CRISPR Design	gRNA design and analysis tool	http://crispr.mit.edu
CRISPR Genome Analyzer	Genome editing experiments tool	http://crispr-ga.net
CRISPR-PLANT	gRNA recognizing tool in plant genome	http://genome.arizona.edu/crispr
CRISPRseek	gRNA design tool	http://bioconductor.org/packages/release/bioc/html/CRISPRseek.html
DNA 2.0 gRNA Design Tool	gRNA design tool	https://dna20.com/eCommerce/cas9/input
E-CRISP	Target DNA identification tool	http://e-crisp-test.dkfz.de/E-CRISP
RGEN Tools	and prediction of off-target sites tool	http://rgenome.net/cas-offinder
sgRNAcas9	gRNA design tool and prediction of off-target sites	http://biootools.com
CRISPR MultiTargeter	multiple gRNA design tool	http://multicrispr.net/
CRISPR-P	gRNA design in plants	http://cbi.hzau.edu.cn/crispr /
AGEseq	Analysis of genome editing by sequencing	https://github.com/liangjiaoxue/AGEseq
Stupar Lab’s CRISPR Design	Target DNA identification tool	http://stuparcrispr.cfans.umn.edu/CRISPR

For fast validation of CRISPR efficiency, transient expression systems have been developed to assess *in vivo* efficiency of CRISPR in a short period of time. The applicability of CRISPR mutagenesis ability for genetic screening in mammalian cell culture to achieve gene inactivation was reported (Koike-Yusa et al., 2014; Zhou et al., 2014; Hartenian and Doench, 2015). The CRISPR-Cas9 screening of protein domains was also applied for detecting cancer drug targets (Shi et al., 2015). In the case plants, the protoplast based transient system has been used to test NHEJ-mediated mutagenesis and HR-mediated gene replacement in Arabidopsis, tobacco, rice, and wheat. Moreover, targeted gene replacements in protoplasts have reached an efficiency of 18.8% to 42.0% in Arabidopsis (Mao et al., 2013; Feng et al., 2014), 9% in tobacco (Li et al., 2013), and 6.9% in rice (Shan et al., 2013a). Other examined transient systems include leaf agro-infiltration in Arabidopsis (Li et al., 2013) and tobacco (Nekrasov et al., 2013; Jiang et al., 2013), embryo transformation in sorghum (Jiang et al., 2013), and suspension cells of wheat (Upadhyay et al., 2013). Mutation detection methods that are widely used include PCR/RE, which can detect disruption of a preserved restriction enzyme site in the targeted sequence, and surveyor assays based on T7 endonuclease or *Cel*I. In the case of *in planta* transformation method, the mutation frequency can reach 89% in Arabidopsis (Mao et al., 2013), and 91.6% in rice (Miao et al., 2013). In addition to point mutations, large deletions in *AtTT4* gene aimed to removal of a 230-bp fragment in Arabidopsis (Mao et al., 2013), and multiplex gene disruptions have been achieved in Arabidopsis (Mao et al., 2013; Li et al., 2013), rice (Miao et al., 2013), and wheat (Upadhyay et al., 2013). Recently, the Cas9-induced mutations were induced in *Hordeum vulgare* (two copies of *HvPM19* gene) and *Brassica oleracea* (*BolC.GA4* gene) with frequencies of 23% and 10% in the first generation, respectively (Lawrenson et al., 2015). More recently, P-CR domain of *CesA4* of white poplar (*Populus alba* L.) was edited using CRISPR/Cas9 to produce nanocellulose with a high efficiency (Nayeri et al., 2022). CRISPR-based screening is the newest genome editing tool for functional analysis of genomes.

The Cas9-VirD2 fusion system, combining Cas9 with VirD2, allows for efficient homology-directed repair (HDR) in genome editing, particularly in rice. This fusion protein combines the DSB generation capability of Cas9 with VirD2’s role in repair template delivery, facilitating HDR. The Cas9-VirD2 system has been used to modify the ACETOLACTATE SYNTHASE (*OsALS*) allele to confer herbicide resistance, alter plant structure, and create in-frame fusions with the HA epitope at the HISTONE DEACETYLASE (*OsHDT*) gene in rice plants. This research demonstrates the potential of the Cas9-VirD2 system for improving agricultural traits and expanding precise genome editing in eukaryotic species (Xu et al., 2020). Additionally, the recent development of PAM-DETECT has enabled the rapid identification of PAMs for Type I CRISPR-Cas systems, allowing for the recognition of a variety of PAMs. Extensive analysis of Type I orthologs has been conducted to characterize self-targeting systems. TXTL-based assays were employed to assess DNA target recognition and transposition by CRISPR-associated transposases (CASTs). Various experiments, including plasmid construction, qPCR analysis, deGFP repression assays, prophage prediction, and transposition studies, were performed to explore different facets of CRISPR effectors and transposons. *In vivo* transposition experiments were conducted using BL21(DE3) competent *E. coli* (Wimmer et al., 2022).

#### 2.5.2 CRISPR/Cas12

CRISPR-Cas12 technology has emerged as a transformative tool for genome editing, enabling precise modifications of DNA or RNA sequences across diverse organisms. This system leverages Cas proteins such as Cas12a and Cas12b, which provide customizable sequence specificity (Fan et al., 2023). Cas12b, in particular, shows significant potential in eradicating human immunodeficiency virus (HIV) through a single guide RNA (gRNA), positioning it as a promising tool for HIV inactivation (Singh et al., 2023). The development of additional Cas variants, including Cas13 and Cas14, has further expanded the versatility of genome editing applications in various organisms (Griffith et al., 2023).

Compared to the widely used CRISPR-Cas9 system, CRISPR-Cas12a exhibits distinct advantages. It provides enhanced target specificity due to its sensitivity to protospacer-adjacent-motif (PAM) distal mismatches (Singh et al., 2023). Additionally, Cas12a simplifies multiplexing by allowing multiple guide RNAs to be expressed from a single transcript, facilitating combinatorial genome perturbations (Griffith et al., 2023). The ability to deliver Cas12a as ribonucleoproteins (RNPs) also minimizes concerns regarding transgene integration and off-target effects (Fang et al., 2023). Moreover, Cas12a’s compact size and capability to produce staggered double-stranded DNA ends after cleavage enhance cellular recombination events (Martin et al., 2023). Unlike Cas9, Cas12a endonucleases exhibit a more selective PAM requirement and generate staggered DNA cuts with 5-7 base overhangs, further improving their precision and applicability (Armstrong et al., 2023).

Initially, CRISPR-Cas12 was deployed for nucleic acid detection, particularly for identifying infectious and zoonotic diseases (Li et al., 2023). Techniques like HOLMES and SHERLOCK utilize the trans-cleavage activity of Cas12 and Cas13, enabling rapid and sensitive nucleic acid detection (Li et al., 2023). These methods employ CRISPR RNA (crRNA) to guide Cas enzymes in binding and cleaving target DNA or RNA, generating detectable signals. To enhance detection sensitivity, these systems are often integrated with pre-amplification techniques such as PCR and isothermal amplification (Yang et al., 2023). For instance, the HOLMESv2 platform has been successfully applied to detect the Japanese encephalitis virus (JEV), showcasing its utility in diagnostic applications (Liu and Dai, 2023).

CRISPR-Cas12 technology has also revolutionized crop breeding by facilitating the precise modification of genetic traits. This includes the development of germplasm with enhanced disease resistance, herbicide tolerance, and improved yield and quality. Examples include resistance to powdery mildew in wheat and tomato, virus resistance in potato and cucumber, and drought tolerance in soybean. Furthermore, CRISPR-mediated genome editing has improved performance traits and nutrient content, such as increasing protein and amylose levels in wheat, enhancing lycopene content and shelf life in tomatoes, and producing cyanide-free cassava and lipoxygenase-free soybeans. The technology has also been used to modify fruit size, grain dimensions, and tiller production in crops like wheat, rice, and tomatoes (**Table 1**). However, many of these engineered crops are still undergoing regulatory review and approval processes (Chen et al., 2017).

Advancements in CRISPR-Cas12 technology continue to broaden its applications in genome editing, diagnostics, and agriculture. With its superior precision, versatility, and scalability, CRISPR-Cas12 is set to play a pivotal role in addressing global challenges in health, food security, and sustainable agriculture.

## 3 Comparison of different molecular editing tools

Many researchers face challenges in selecting the most suitable system due to the increasing availability of molecular tools for reverse genetics approaches. Each genome editing technology has its strengths and limitations, choosing the right tool is highly dependent on the specific experimental design. **Table 3** provides a comprehensive comparison of the remarkable new genome editing tools.

**Table 3:** Comparison among the genome engineering tools.

	RNAi	Meganuclease	ZFNs	TALEN	CRISPR/Cas9	CRISPRi	CRISPRa
Loss-of-function mechanism	Post-transcriptional RNA degradation	Frame shift DNA mutation	Frame shift DNA mutation	Frame shift DNA mutation	Frame shift DNA mutation	Repression of transcription	Activation of transcription
Result	Reversible knockdown	Permanent knockout	Permanent knockout	Permanent knockout	Permanent knockout	Reversible knockdown	Reversible activation
Transgenes	si/shRNA	Meganuclease	ZFN	TALEN	Cas9 nuclease	dCas9-KRAB	dCas9-VP64
					sgRNA	sgRNA	sgRNA
Guiding sequence	si/shRNA		DBD	DBD	sgRNA	sgRNA	sgRNA
Required sequence information	Transcriptome	Transcriptome	Transcriptome	Transcriptome	Transcriptome	Annotated TSS	Annotated TSS
Off-target space	Transcriptome		Genome; requires FokI dimerization	Genome; requires FokI dimerization	Genome; cuts as monomer	Window around TSS	Window around TSS
Transcript variants	All variants via conserved region	All variants via conserved region	All variants via conserved region	All variants via conserved region	All variants via conserved region	Only variants from the same TSS	Only variants from the same TSS

**Figure 6:**
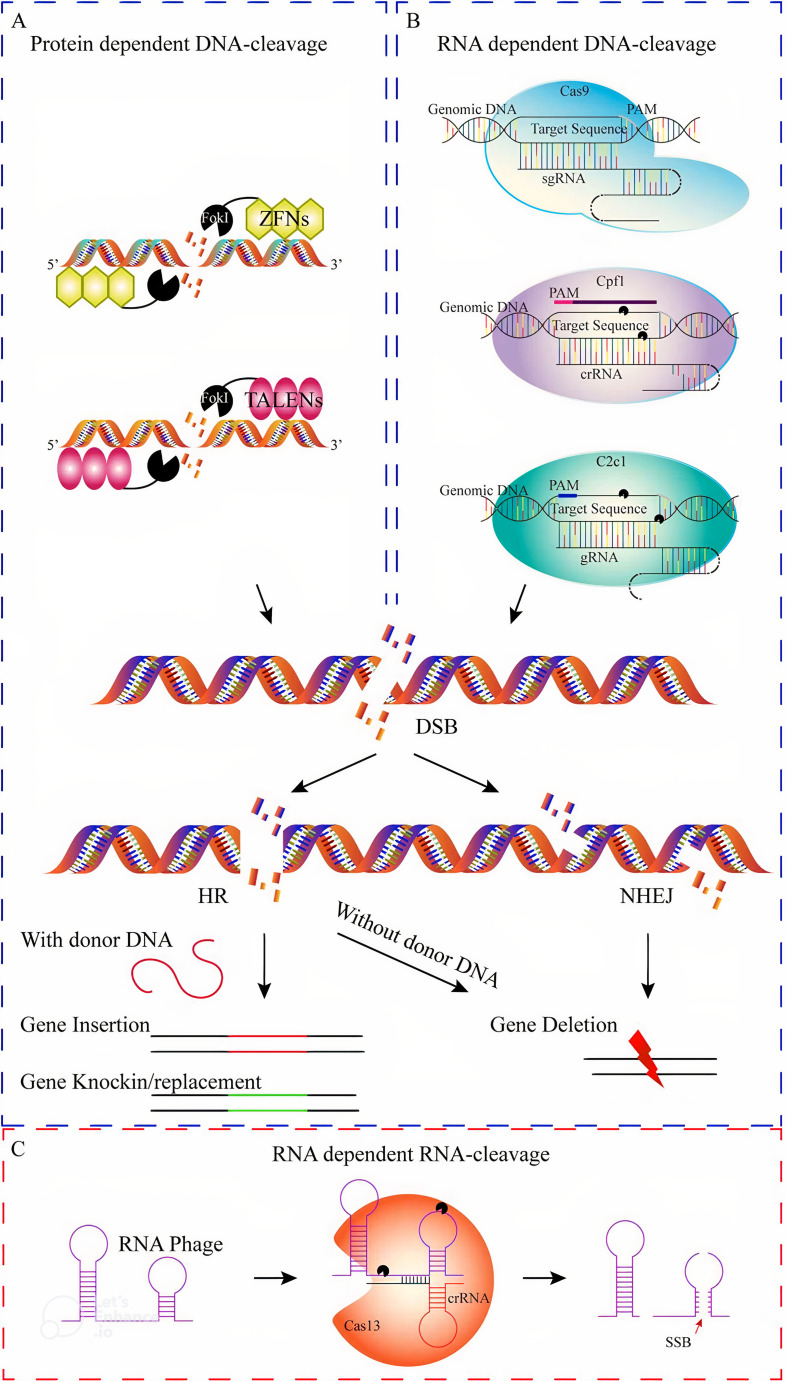
Various genome editing systems used in plants can be compared based on their mechanisms. These systems include site-specific genome editing tools (GETs) such as protein-dependent DNA cleavage systems **(A)**, RNA-dependent DNA cleavage systems **(B)**, and RNA cleavage systems **(C)**. Protein-dependent DNA cleavage systems, such as ZFNs and TALENs, utilize sequence-specific proteins to guide the *FokI* nuclease to the desired DNA site. Similarly, TALENs consist of two sequence-specific TALEN proteins that guide the *FokI* nuclease. On the other hand, RNA-dependent DNA cleavage systems **(B)**, such as CRISPR/Cas9, CRISPR/Cpf1, and CRISPR/C2c1, induce double-strand breaks (DSBs) using the Cas9 nuclease and single-guide RNA. The repair of DSBs can occur through non-homologous end joining (NHEJ) or homologous recombination (HR), with NHEJ often leading to gene knock-out mutations and HR resulting in gene knock-in or replacement. In contrast, RNA-dependent RNA cleavage systems **(C)**, like single-strand break (SSB), can cause random or targeted mutations through error-prone NHEJ or error-free HR, respectively. These genome editing approaches involve the insertion, deletion, or replacement of specific DNA sequences (Ahmad et al., 2020).

**Figure 7:**
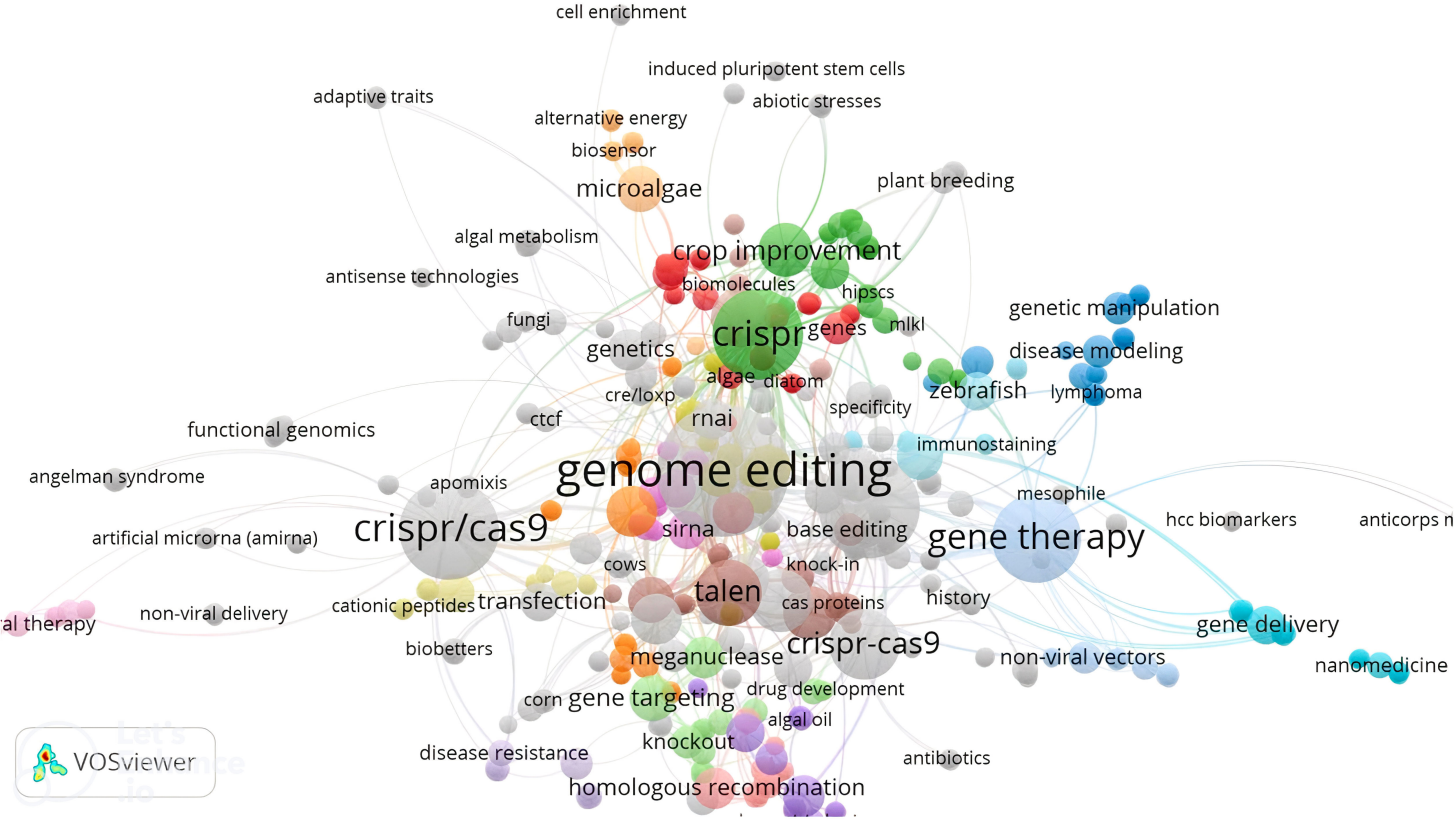
The analysis of different genome editing techniques, including CRISPR/Cas, TALENs, ZFNs, RNAi, and meganuclease, across published literature from 2013 to 2023 reveals that CRISPR is the most extensively studied method, with the largest number of articles. The remaining four methods are ranked below CRISPR in terms of publication frequency.

In **Figure 6**, a comparison is made between the mechanisms and effectiveness of different genome editing systems, such as zinc finger nucleases (ZFNs), transcription activator-like effector nucleases (TALENs), and CRISPR/Cas9. While ZFNs and TALENs use protein motifs for target identification, CRISPR/Cas9 utilizes RNA-DNA recognition to induce double-strand breaks. The CRISPR/Cas system, particularly with the enhancements in CRISPR/Cas12, has become a more precise and powerful tool. Despite its advantages, further improvements are still needed. A comprehensive analysis of various genome editing tools was conducted using the VOSviewer online search tool (version 1.6.20; Van and Waltman, 2010), with the results displayed in **Figure 7**. The relationship among the five genome editing methods is clearly outlined. Research related to CRISPR is the most extensive, highlighting its significance in the field. Following CRISPR, TALEN emerges as the second most prominent method, while RNAi and meganucleases are grouped based on article volume and they occupy two next ranks. Conversely, articles associated with ZFN were relatively scarce, positioning ZFN at the bottom of the list. This comparison offers valuable insights into the relative prevalence and adoption of these genome editing techniques over the analyzed period.

## 4 Future perspectives

Genome editing technologies offer tremendous potential for future applications across diverse fields. In regenerative medicine, genome editing can be employed to generate immune-evasive pluripotent stem cell-derived somatic cells for transplantation, enabling better regulation of immune responses and enhancing therapeutic outcomes (Colella et al., 2023). These tools can also play a crucial role in *in vitro* disease modeling and regenerative medicine, facilitating the understanding of genetic underpinnings and the development of treatments aimed at correcting genetic mutations and curing diseases permanently (Sackett et al., 2022). In agriculture, genome editing techniques such as CRISPR/Cas9 and base editing enzymes offer the ability to rapidly produce improved crop varieties with desired traits. These technologies enable precise nucleotide modifications, removal of specific DNA segments, insertion of foreign DNA fragments, and even epigenetic changes (Kues et al., 2022; Singh et al., 2023). Over the decades, significant advancements have been made in sequence-specific nuclease technologies. A key challenge with meganuclease technology is the substantial engineering required to develop enzymes with unique DNA recognition capabilities, due to the close relationship between cleavage and binding activity in meganucleases. To address this, two approaches have emerged. One involves exploring a wide range of naturally occurring endonuclease families (Taylor and Stoddard, 2012), which expands the pool of meganucleases with unique specificity for genome editing applications. Another strategy combines the flexibility of the TALE DNA-binding domain with the nuclease domain of meganucleases to create a hybrid nuclease structure (Beurdeley et al., 2013; Boissel et al., 2014; Kleinstiver et al., 2014).

Other nucleases, such as ZFNs and TALENs, face similar challenges, including germline transmission, off-target cleavage, and chromatin accessibility. However, ZFNs offer several advantages over other nuclease systems. For instance, ZFNs can be delivered directly to cells through protein delivery, whereas TALENs and Cas9 proteins, due to their larger size and different protein characteristics, do not have the same delivery capacity (Gaj et al., 2013). Additionally, TALENs and CRISPR/Cas9 systems are derived from bacterial proteins, which may lead to stricter regulations for genetically modified organisms (GMOs) generated using these technologies.

When compared to meganucleases and TALENs, ZFNs and CRISPR/Cas systems are generally easier to manipulate and offer more flexibility in target site selection. This allows researchers to choose from a broad range of options for modifying genomes according to specific needs. While TALENs and CRISPR/Cas are increasingly popular, meganucleases retain certain advantages, such as their compact size and high precision, making them particularly useful for therapeutic applications. Unlike meganucleases, ZFNs and TALENs require dimerization to activate the *FokI* nuclease domain for DNA cleavage. As a result, a pair of monomers must be created and delivered for each target site, which can limit the use of viral vectors for ZFN and TALEN delivery in specific cell lines. Furthermore, CRISPR/Cas9 technology poses a higher risk of off-target cleavage when paired with single-guide RNAs, which requires the use of nicking enzymes to achieve a level of specificity comparable to other major genome editing platforms (Stoddard, 2014; Boissel et al., 2014).

For over a decade, RNA interference (RNAi) technology has been the primary method for studying gene function by reducing or disrupting normal gene expression in eukaryotes (Boettcher and McManus, 2015). However, RNAi has raised concerns about off-target effects of small interfering RNAs (siRNAs), which are dose-dependent and often lead to dominant phenotypes, complicating gene function studies (Wang et al., 2009; Franceschini et al., 2014). In contrast, CRISPR interference (CRISPRi) has shown to provide more effective gene knockdowns and significantly stronger loss-of-function phenotypes compared to RNAi (e.g., six out of eight sgRNAs were able to reduce GFP levels by at least 75%) (Gilbert et al., 2013, 2014). However, CRISPRi is also not without its limitations. The effectiveness of the Cas9 nuclease in targeting genes is influenced by the accessibility and location of the transcription start site (TSS), which can be obstructed by chromatin structure (Kuscu et al., 2014; Wu et al., 2014). Moreover, many genes have alternative transcripts, some with TSSs that are located far apart (Sandelin et al., 2007).

For gain-of-function studies in mammalian cells, CRISPR activation (CRISPRa) has become a common tool. Typically, this involves overexpressing transgenic open reading frames (ORFs or cDNAs) in target cells. The success of CRISPRa depends on the optimal targeting of the region upstream of the TSS, usually spanning 400 to 500 nucleotides. However, this region overlaps with the target space for CRISPRi, which spans from 0 to +500 nucleotides downstream of the TSS, creating a challenge in distinguishing between the two approaches. As with CRISPRi, knowing the precise location of the TSS is essential for effective CRISPRa-mediated gene activation, and controlling the activation of multiple transcripts from the same TSS individually remains difficult (Gilbert et al., 2014; Konermann et al., 2015).

Given the rapid development of CRISPR-based technologies, it raises the question of whether RNAi-based tools are becoming obsolete. While RNAi remains a simpler and faster approach for generating hypomorphic knockdowns, especially because it does not require the introduction of additional components such as Cas9 proteins or tracrRNA, CRISPRi offers more precise gene regulation. RNAi also does not target TSSs and can be used in species with only transcriptomic data, as noted by Kodzius et al. (2006). Moreover, RNAi can target conserved sequences among transcript variants or gene family members, allowing a single si/shRNA to target multiple transcripts regardless of TSS location, whereas CRISPRi-based tools face limitations in this regard. Additionally, RNAi operates in the cytoplasm, which means its accessibility is not hindered by chromatin structure, a challenge that CRISPR-based technologies often face (Kuscu et al., 2014; Wu et al., 2014).

Off-target genome editing refers to unintended DNA modifications caused by inaccurate gRNA targeting or gRNA-independent mechanisms (Jin et al., 2019). To address this issue, two main approaches have been developed: methods for detecting off-target effects and strategies to enhance editing precision in the CRISPR system. Bioinformatics tools such as CasOFFinder (http://www.rgenome.net/cas-offinder/) and CCTop (https://crispr.cos.uniheidelberg.de), along with techniques like SELEX, IDLV capture, Guide-seq, HTGTS, BLESS, Digenome-seq (Koo et al., 2015), and DISCOVER (Wienert et al., 2019), have been created to tackle off-target effects. Researchers must choose the most appropriate analytical tool depending on their specific research goals, considering the strengths and limitations of each method. Moreover, progress in engineering Cas9 proteins with improved target specificity, such as eSpCas9 (Slaymaker et al., 2016), HF-Cas9 (Kleinstiver et al., 2016), HypaCas9 (Chen et al., 2017), and Sniper Cas9 (Lee et al., 2018), has shown significant reductions in off-target effects while maintaining high on-target activity. Additionally, the engineering of gRNAs has also contributed to improving specificity. However, concerns remain regarding off-target mutations, such as those observed in rice with cytosine, but not adenine, base editors (Jin et al., 2019), emphasizing the need for further refinement of these tools. Furthermore, the safety and commercialization of genome-edited organisms are significant considerations. Since genome-edited plants do not contain foreign genetic material, transgene-free systems like TKC and VIGE would not classify these plants as transgenic, which could facilitate their commercialization. Nonetheless, acceptance of genome-edited crops remains contentious, with some countries embracing their cultivation, while others continue to debate the issue. As these technologies advance, the editing of multiple genes in crops is expected to become more common, allowing for enhanced characteristics in desired cultivars (Ahmad et al., 2020).

## 5 Ethical and regulatory considerations

In the past biennium, approximately 190 million hectares of genetically modified (GM) crops were cultivated across 26 countries, including 21 developing nations and five industrialized ones. Brazil, Argentina, and India rank among the top five countries with the largest areas dedicated to biotechnology crop production, collectively accounting for 54% of the increase in developing nations (Turnbull et al., 2021).

Historically, the product-centric model has been more prevalent in the United States, Canada, and other American countries, where genetically modified organisms (GMOs) are regarded as comparable to those developed through traditional selection methods (Medvedieva and Blume, 2018). In this model, such organisms fall under existing legal frameworks that aim to mitigate potential risks to human health and the environment, rendering additional regulatory measures unnecessary (Turnbull et al., 2021). In contrast, the European Union has traditionally supported a process-oriented approach (Medvedieva and Blume, 2018). This perspective acknowledges gene-modification technologies as distinct and fundamentally innovative, thus justifying the need for specialized regulatory frameworks (Duensing et al., 2018). Several GM plant products, including maize, soybeans, oilseed rape, and cotton, are already governed by EU legislation. The legislation allows for various methods depending on risk assessment protocols but excludes approaches such as mutation breeding, which were used prior to the enactment of the directive in 2001 (Van der Meer et al., 2023). Process-based regulations in the EU could extend to CRISPR-Cas9 technology if it is categorized as a variant of conventional genetic engineering resulting in GMO production (Zhang et al., 2020).

The CRISPR-Cas9 system has been successfully used to modify over 25 plant species and 100 genes, producing various desirable traits in key crops (Manghwar et al., 2019). Despite its proven effectiveness in enhancing crop genetic traits (Zhang et al., 2020), debates regarding the pros and cons of utilizing CRISPR-Cas technology for agricultural food production led to a landmark ruling by the Court of Justice of the European Union (CJEU) in July 2018 (Purnhagen and Wesseler, 2021). The CJEU decision classified CRISPR-Cas as a technique subject to the ‘mutagenesis exception’ outlined in Appendix 1 B of the Genetically Modified Organism (GMO) directive in the preliminary reference case Confédération Paysanne (C-528/16) (Siebert et al., 2022). Consequently, new plant breeding techniques (NPBTs) explicitly excluded ‘oligonucleotide-directed mutagenesis’ from the mutagenesis exemption, reclassifying the technology as derived from GMOs (Urnov et al., 2018). This legal classification creates challenges for biotechnology companies using CRISPR-Cas technology, resulting in increased regulatory hurdles and financial costs, such as the need for marketing authorization and adherence to product labeling requirements (Turnbull et al., 2021). Thus, the adoption and integration of CRISPR-Cas as an advanced gene-editing tool remains a contentious global issue, particularly among researchers and stakeholders directly involved in the technology.
